# Study of Alginate-Supported Ionic Liquid and Pd Catalysts

**DOI:** 10.3390/nano2010031

**Published:** 2012-01-11

**Authors:** Claire Jouannin, Chloë Vincent, Isabelle Dez, Annie-Claude Gaumont, Thierry Vincent, Eric Guibal

**Affiliations:** 1 Ecole des Mines d’Alès, Laboratoire Génie de l’Environnement Industriel, Equipe Interfaces Fonctionnalisées pour l’Environnement et la Sécurité, 6, Avenue de Clavières, F-30319 ALES Cedex, France; Email: claire.jouannin@ensicaen.fr (C.J); Email: chloe_vincent@live.fr (C.V); Email: thierry.vincent@mines-ales.fr (T.V); Email: eric.guibal@mines-ales.fr (E.G); 2 Laboratoire de Chimie Moléculaire et Thioorganique, UMR CNRS 6507, INC3M, FR 3038, ENSICAEN & Université de Caen, F-14050 Caen, France; Email: isabelle.dez@ensicaen.fr (I.D); Email: annie-claude.gaumont@ensicaen.fr (A.C.G)

**Keywords:** alginate, ionic liquid, highly porous monolith, palladium, 4-nitroaniline, catalytic hydrogenation

## Abstract

New catalytic materials, based on palladium immobilized in ionic liquid supported on alginate, were elaborated. Alginate was associated with gelatin for the immobilization of ionic liquids (ILs) and the binding of palladium. These catalytic materials were designed in the form of highly porous monoliths (HPMs), in order to be used in a column reactor. The catalytic materials were tested for the hydrogenation of 4-nitroaniline (4-NA) in the presence of formic acid as hydrogen donor. The different parameters for the elaboration of the catalytic materials were studied and their impact analyzed in terms of microstructures, palladium sorption properties and catalytic performances. The characteristics of the biopolymer (proportion of β-D-mannuronic acid (M) and α-L-guluronic acid (G) in the biopolymer defined by the M/G ratio), the concentration of the porogen agent, and the type of coagulating agent significantly influenced catalytic performances. The freezing temperature had a significant impact on structural properties, but hardly affected the catalytic rate. Cellulose fibers were incorporated as mechanical strengthener into the catalytic materials, and allowed to enhance mechanical properties and catalytic efficiency but required increasing the amount of hydrogen donor for catalysis.

## 1. Introduction

Heterogeneous catalysis is generally preferred to homogeneous catalysis, at industrial scale, since the immobilization of catalytic species on a solid support improves their recovery and recycling: this is especially important when catalytic metals are strategic or precious metals or when they consist of complex metal-ligand systems [1]. The critical parameters for supported catalysis (compared to homogeneous catalysis) are the diffusion properties (accessibility and availability of the active species), the stability of the catalytic metals (release and recovery), and the selectivity (chemo- and stereo-selectivity). A system associating the advantages of both homogeneous and heterogeneous catalysis would be promising for industrial development of catalytic processes by offering high activity, selectivity, easy recycling of the catalyst, and ready separation of the substrates and products. The immobilization of a catalyst contained in a solvent phase on the surface of a highly porous support may lead to such a system. This concept allows minimizing the amount of solvent and mass transfer resistance, optimizing surface exchanges and catalytic recovery.

As an alternative to conventional solvents, new media based on ionic liquids (ILs) have recently been developed. These “alternative solvents” are very interesting substituents for volatile solvents (less hazardous, easy product and catalyst separation, *etc.*) [2,3,4]. However, due to their high cost, there are some limitations to their wide spread use in industry for homogeneous catalysis. The immobilization of ILs on a suitable support is then a very interesting alternative for the development of supported solvent catalytic systems—referred to as supported ionic liquid catalysts (SILC) [5,6,7].

As the first examples of SILC have been designed with silica supports [8,9], an increasing attention is now paid to the use of renewable polymeric supports and more specifically biopolymers such as cellulose, chitosan or alginates [10,11,12]. Alginates are polysaccharides, mainly extracted from brown seaweeds. They are linear polymers composed of β-D-mannuronic acid (M) and α-L-guluronic acid (G), with various M/G ratios depending on the source of alginate. Alginates are soluble in water and are able to form gels with divalent cations such as Ca^2+^ (calcium alginate), or with lowering of the pH (alginic acid) [13].

Polysaccharides have been previously used as support for the immobilization of catalytic metals due to their own affinity for metal ions that can be bound by complexation, chelation, ion exchange mechanisms, or by specific reductive precipitation [14,15]. The resistance to mass transfer revealed a key parameter for the control of catalytic performance [16], necessitating the design of alternate conditionings that minimize the diffusion resistance (such as hollow fibers) [17]. These alternate conditionings show direct catalytic effects and can be used as supports, especially when designed under highly porous forms, by CO_2_ supercritical drying for example [18,19,20,21]. These biopolymers have also been used for the immobilization of solvent extractants and ionic liquids for manufacturing specific resins or extractant impregnated resins (EIR) [22]. The combination of the biopolymer with the IL allows preparation of supports for the binding of a wide range of metals, including those used for catalysis (such as Pd) [23]. The encapsulation of ILs offers an alternative to the surface-immobilization of ILs in conventional biopolymer-SILC systems [10,11], keeping in mind that the accessibility to catalytic sites and the mass transfer properties remained major challenges.

In a previous work, new catalytic systems using alginate to immobilize ILs and palladium, in the form of highly porous monoliths (HPMs) were studied for the hydrogenation of 4-nitroaniline [24]. The present work focuses on the influence of the parameters of elaboration of the catalytic materials. The different parameters studied were the M/G ratio of alginate, the biopolymer concentration, the freezing temperature, the type of ionic liquid, the concentration of porogen agent, the type of coagulating agent, and the presence of cellulose fibers as natural mechanical strengthener. The catalytic activity of the different materials were compared using the hydrogenation of 4-nitroaniline (4-NA) into *p*-phenylenediamine in the presence of formic acid as the hydrogen donor as a model reaction [24].

This study presents, in a first part, the characterization of the catalytic materials by palladium sorption studies, by ICP-AES analysis to determine the amounts of IL and Pd in the materials, and by SEM-EDX analysis to identify the distribution of elements within the materials, the morphology of the materials, and their mechanical resistance to compression (non-standardized test). In a second part, the catalytic properties of the different materials are reported for the hydrogenation of 4-NA. Finally, a summary of the different parameters that might be considered for specific applications of the materials is presented.

## 2. Results and Discussion

The catalytic materials obtained from the different batches (depending on the M/G ratio of alginate, the biopolymer concentration, the freezing temperature, the type of ionic liquid, the concentration of NaHCO_3_ as porogen agent, the type of coagulating agent, and the presence of cellulose fibers as mechanical strengthener) are reported in Table 1.

**Table 1:** Conditions for the elaboration of alginate-supported ionic liquid (IL) materials.

Ref.	IL	(%)	type	(w/w)	Coagulating agent *	T (°C)	Cellulose
A1	111	2	A	4	HCl	−78 °C	No
A2	111	2	A	4	HCl	−20 °C	No
A3 ^(a)^	111	2	A	4	CaCl_2_	−78 °C	No
A4 ^(a)^	101	2	LF-240D	4	CaCl_2_	−78 °C	No
A5 ^(a)^	101	2	LF-200S	4	CaCl_2_	−78 °C	No
*A6 ^(b)^*	*111*	*3*	*A*	*4*	*HCl*	*−78 °C*	*No*
*A7 ^(b)^*	*111*	*3*	*A*	*8*	*HCl*	*−78 °C*	*No*
*A8 ^(b)^*	*111*	*2*	*A*	*8*	*HCl*	*−78 °C*	*No*
A9	111	2	A	2	HCl	−78 °C	No
A10	111	2	A	4	HCl	−78 °C	Yes

Type of IL: Cyphos IL‑101 (tetradecyl(trihexyl)phosphonium chloride) or Cyphos IL-111 (tetradecyl(trihexyl)phosphonium tetrafluoroborate); Gelatin/IL ratio: 2 w/w; Alginate type: Acros (A), Protanal LF-240 D and Protanal LF-200S; * Coagulating agent: 0.5 M HCl or 0.4 M CaCl_2_; ^(a)^ poor mechanical resistance to compression; ^(b)^ poor stability in water.

### 2.1. Characteristics of Materials

#### 2.1.1. Materials with Low Stability in Water

Among the different catalytic materials, some samples (**A6–8**) showed poor stability in water (dissolving in water after 24 h).

The catalytic materials prepared with an alginate concentration of 3% (**A6** and **A7** samples) dissolved in water after 24 h, contrary to the standard samples prepared with an alginate concentration of 2% (**A1–5** and **A9–10** samples). Thus, a biopolymer solution of 2% should be considered as a suitable concentration for the elaboration of the catalytic materials.

Similarly, the preparation of a catalytic material with a high Alginate/NaHCO_3_ mass ratio of 8 led to a material poorly stable in water (**A7** and **A8** samples), whereas the use of an Alginate/NaHCO_3_ mass ratio of 4 or 2 in other samples allowed to obtain stable materials. Therefore, the Alginate/NaHCO_3_ ratio of 8 was not used anymore for the elaboration of catalytic materials.

The poor stability of **A6–8** samples limited the possibility to use them for catalytic reactions (Table 1).

#### 2.1.2. Pd(II) Sorption Properties

Palladium sorption studies were carried out in order to evaluate the influence of the freezing temperature or the coagulating agent on Pd(II) sorption capacities of the alginate-supported IL materials. Indeed, the freezing temperature might influence the porosity of the materials, thus being likely to influence sorption kinetics. Similarly, the coagulating agent might have an influence on the accessibility of binding groups for Pd(II) sorption.

The uptake kinetics and sorption isotherms of Pd(II) (at pH 1) were compared for selected alginate-supported IL materials: **A1** (freezing at −78 °C and HCl gelation), **A2** (freezing at −20 °C and HCl gelation) and **A3** (freezing at −78 °C and CaCl_2_ coagulation) (Figure S1 and S2, Supplementary Information). Alginate-supported IL materials had good sorption performance for Pd(II). Maximum sorption capacities were similar for **A1**, **A2** and **A3** samples, close to 90 mg Pd g^−1^. It should be noted that in this Pd(II) sorption study, the palladium was not reduced.

The uptake kinetics showed for **A1** and **A3** samples that the equilibrium value was in the same order of magnitude for both materials, but the time required to reach the equilibrium was substantially increased when CaCl_2_ was used for the coagulation of the materials (48 h *versus* 10 h, for the materials prepared with HCl gelation). Thus, CaCl_2_ coagulation caused limitations in mass transfer during Pd(II) sorption on alginate-supported IL materials. On the other hand, no influence of the freezing temperature was observed by the comparison of Pd(II) sorption isotherms of **A1** and **A2** samples.

#### 2.1.3. Element Analysis of the Catalytic Materials

The mineralization of catalytic materials, followed by ICP-AES analysis of the solutions, allowed quantifying the amounts of IL and Pd immobilized on the materials (Table 2).

The results showed that the IL immobilization in the different materials varied from 22.7 to 30.8 mg IL g^−1^. The materials prepared with CaCl_2_ coagulation (**A3**, **A4** and **A5** samples) contained slightly lower amounts of IL than the materials prepared with HCl gelation (**A1**, **A2** and **A9** samples). Thus, HCl gelation was favorable to immobilize higher IL quantities in the materials. Even if the material prepared with the inclusion of cellulose fibers was also prepared with HCl gelation (**A10** sample), it presented slightly lower IL content compared with standard materials. This could be easily explained by the presence of cellulose fibers in the material, that contributed to decrease the fraction of alginate in the material, thus, also decreased the amount of IL immobilized on the material.

**Table 2:** Characteristics of selected catalytic materials.

Material	(mg Pd g^−1^)	(mg LI g^−1^)	(mol/mol)
A1	62.8 ± 2.2	27.8 ± 2.2	0.66
A2	54.5 ± 4.3	30.8 ± 2.0	0.51
A3	46.9 ± 2.4	22.7 ± 1.5	0.6
A4	20.7 ± 1.0	25.0 ± 0.7	0.24
A5	15.3 ± 1.1	24.5 ± 1.6	0.18
A9	22.0 ± 1.5	29.5 ± 0.9	0.22
A10	18.9 ± 1.3	24.7 ± 0.5	0.22

More significant differences were observed regarding the amount of Pd immobilized on the materials: from 15.3 to 62.8 mg Pd.g^−1^. The highest Pd amounts were accumulated on **A1**, **A2** and **A3** samples (about 2 to 3 times higher than for other samples). These differences in the quantity of Pd immobilized in alginate-supported IL materials could not be related to a specific parameter in the elaboration of the materials. The Pd/IL molar ratio was much higher for **A1**, **A2** and **A3** samples (in the range 0.5–0.66) compared to **A4**, **A5**, **A9** and **A10** samples (in the range 0.18–0.24). In a former work dealing with the sorption of Pd by alginate beads containing IL [23], the interaction of Pd chloroanionic complex (PdCl_4_^2^^−^) with Cyphos IL-101 ([R_3_R’P^+^][Cl^−^]) was suspected to follow a stoichiometric ratio between Pd and IL of 1:2 [23]. Thus, the molar ratio (Pd/IL) at saturation of the alginate-supported IL materials was expected to be close to 0.5. In our case, the molar ratio of 0.6–0.7 probably meant that other reactive groups might contribute to the sorption of Pd(II) at pH 1, or that secondary phenomena (such as Pd agglomeration) might occur around initially bound Pd.

The different amounts of Pd immobilized in each catalytic material will be taken into consideration for the study of the catalytic efficiency of the materials.

#### 2.1.4. Morphology of the Catalytic Materials

SEM observations were performed on the cross-section of the catalytic materials in order to determine the structure of the HPMs. The observation of SEM photographs at different growing magnitude allowed comparison of the texture and the organization of material porosity (See Supplementary Information, Figures S3–9).

The structure of catalytic materials showed the morphology of a foam sponge with long plane alveoli. However, the aspect of the porous network significantly changed with the process of elaboration of the catalytic materials.

The influence of the freezing temperature (−78 °C *versus* −20 °C) was studied by comparison of **A1** and **A2** samples (Figures S3 and S4). The freezing temperature is a parameter known to affect the structure of the pores in biopolymer scaffolds [25,26,27,28]. In the elaboration of our materials, it was noted that decreasing the freezing temperature led to less-oriented and less-opened structures. Yuan *et al*. reported significant changes in the structure of alginate and chitosan when both the temperature of the freezing step and the cooling speed were changed: fast cooling induced non-simultaneous nucleation and generated directional pore structures, while slow cooling led to simultaneous nucleation and the production of uniform and isotropic pores [26]. With the experimental conditions selected for this study, the fast-cooling conditions corresponded to the freezing temperature −78 °C, while −20 °C corresponded to the slow cooling conditions. Thus, the freezing temperature had an influence on the porosity of the catalytic materials, and a more homogeneous porosity was obtained at the freezing temperature −20 °C (**A2** sample) compared to −78 °C (**A1** sample).

The coagulating agent slightly influenced the structure of the materials. In the case of the catalytic materials prepared with the Acros alginate, the material coagulated with CaCl_2_ (**A3** sample, Figure S5) showed a less organized structure (mashed ribbons) compared with the material prepared with HCl gelation (**A1** sample, Figure S3). However, in the case of the catalytic materials prepared with the two other alginates, Protanal LF-240D (**A4** sample, Figure S6) and LF-200S (**A5** sample, Figure S7), the coagulation with CaCl_2_ led to structures similar to those obtained with HCl gelation for the catalytic material prepared with Acros alginate (**A1** sample, Figure S3). Thus, the coagulation of alginate in the form of calcium alginate (with CaCl_2_) or the gelation in the form of alginic acid (with HCl) has little influence on the porosity observed by SEM.

The influence of the concentration of the porogen agent NaHCO_3_ on porosity was also evaluated by SEM observations. Changing the mass ratio between alginate and NaHCO_3_ from 4 to 2 in samples **A1** and **A9**, respectively, hardly changed the structure of the porous network (**A1** sample, Figure S3 and **A9** sample, Figure S8). Thus, the impact of the concentration of the porogen agent NaHCO_3_ on the structure of the catalytic material was not observed on SEM photographs.

On the other hand, the addition of cellulose fibers as mechanical strengthener in the catalytic material, led to reduced pore size (**A10** sample, Figure S9) when compared to standard catalytic material (**A1** sample, Figure S3). The cellulose fibers are supposed to arm the polymer gel, limiting the possible deformation of the network during freezing and gelation.

In addition to porosity analysis, the SEM photographs also allowed to observe the impact of the parameters used for the elaboration of catalytic materials on the internal structures of the HPMs and the distribution of ionic liquid and Pd. Indeed, in SEM observations with backscattered electrons, heavy elements (high atomic numbers) appear by phase contrast, as white spots. Palladium was thus expected to be located in the white areas observed in SEM photographs. The IL was immobilized as encapsulated drops at the internal surface of porous network (**A1–2** and **A9–10** samples; Figures S3–4 and S8–9), and Pd was localized at the surface and at the interface of the IL/alginate “globules”. In the case of the catalytic materials prepared with CaCl_2_ coagulation (**A3–5** samples, Figures S5–7), the internal structure was less resolved (the globules were less easy to identify) and Pd element appeared at the surface of the alginate matrix in the form of small aggregates. In conclusion, the nature of alginate (calcium alginate or alginic acid) has little influence on the porosity of the catalytic material but has an influence on the way palladium was fixed on the material.

#### 2.1.5. Distribution of Elements

**Figure 1:**
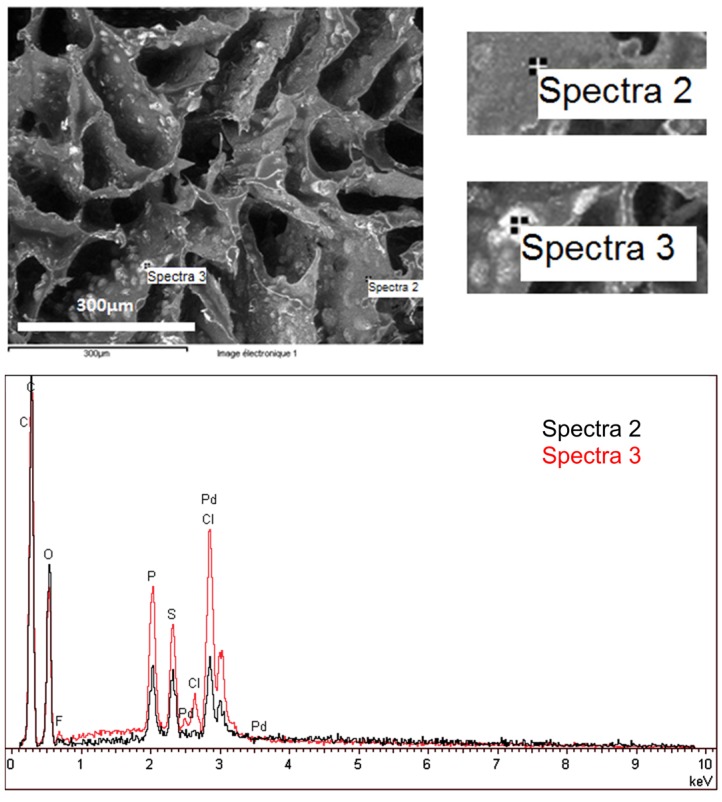
SEM-EDX analysis of specific zones of **A1** catalytic material: focus on a non-specific zone (grey area, Spectra 2) and on an IL vesicle (white area, Spectra 3).

**Figure 2:**
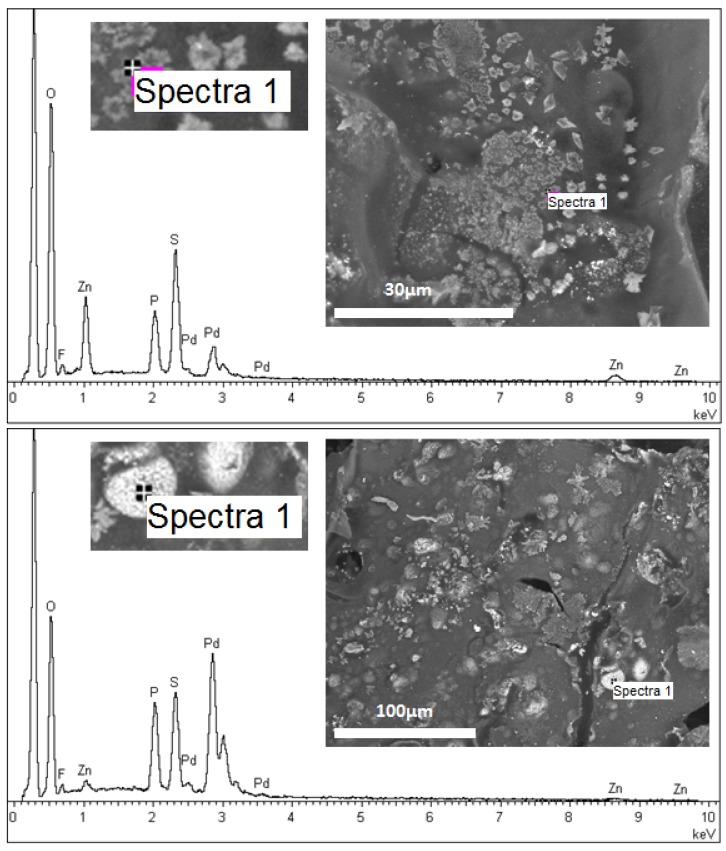
SEM-EDX analysis of specific zones of **A3** catalytic material: focuses on white areas (Spectra 1).

The distribution of chemical elements within the catalytic material was determined by SEM-EDX analyses. As described previously, the nature of alginate (calcium alginate or alginic acid) was supposed to influence Pd binding in the material. Thus, SEM-EDX analyses were performed on two catalytic materials, prepared with HCl gelation (**A1** sample, Figure 1), and with CaCl_2_ coagulation (**A3** sample, Figure 2).

In the case of the catalytic material prepared with HCl gelation (**A1** sample, Figure 1) two zones were analyzed: a white spot (Spectra 3) and a grey area (Spectra 2). On the grey zone (Spectra 2), the analysis detected the organic material with small amounts of Pd, P and S elements. The presence of P (tracer of the IL cation) and Pd elements confirmed that the palladium was bound to the IL. S element might be explained by the procedure used for Pd(II) reduction (reaction of sulfuric acid with Zn powder). On the white spot (Spectra 3), F and Cl elements were detected, in addition to the previous elements. F, present in small amounts, was representative of Cyphos IL-111 anion, and the presence of Cl might be explained by an incomplete Pd reduction. The intensity of elements (P, Pd, S, Cl) appeared to be higher on the white spot (Spectra 3) than in the grey area (the Spectra 2). The intensities that were measured should be considered as indicative data (non-quantitative) since the focus of electron beam did not allow restricting the analysis to a fine spot, but analyzed a small volume around the spot. However, the difference in intensities between the two spectra was explained by the major presence of IL in vesicles containing also palladium. Indeed, a small fraction of IL was included in the alginate matrix but the highest IL loading was localized in the vesicles.

In the case of the catalytic material prepared with CaCl_2_ coagulation (**A3** sample, Figure 2), two SEM-EDX analyses were performed on white areas. On the dense spots, the signals for Pd and P elements were much higher than for the grey surface. This analysis confirmed the presence of IL and Pd on white areas compared to the alginate matrix on grey surface. In this analysis on a white area, the signal of Zn was significantly increased. The strong Zn signal might be explained by the binding of Zn on the catalytic material: the *in situ *production of hydrogen was associated to the oxidation of Zn powder and the release of Zn(II), which can form anionic complexes with chloride ions exchanged during Pd(II) sorption. These chloroanionic species could bind to the phosphonium cations of the IL. The fraction of Zn(II) bound on vesicles was much lower because of the greater affinity of the IL for tetrachloropalladate species [23].

The SEM-EDX analysis confirmed that Pd and IL are included in the alginate matrix, by immobilization of Pd in IL vesicles within the alginate material.

#### 2.1.6. Mechanical Properties

**Figure 3:**
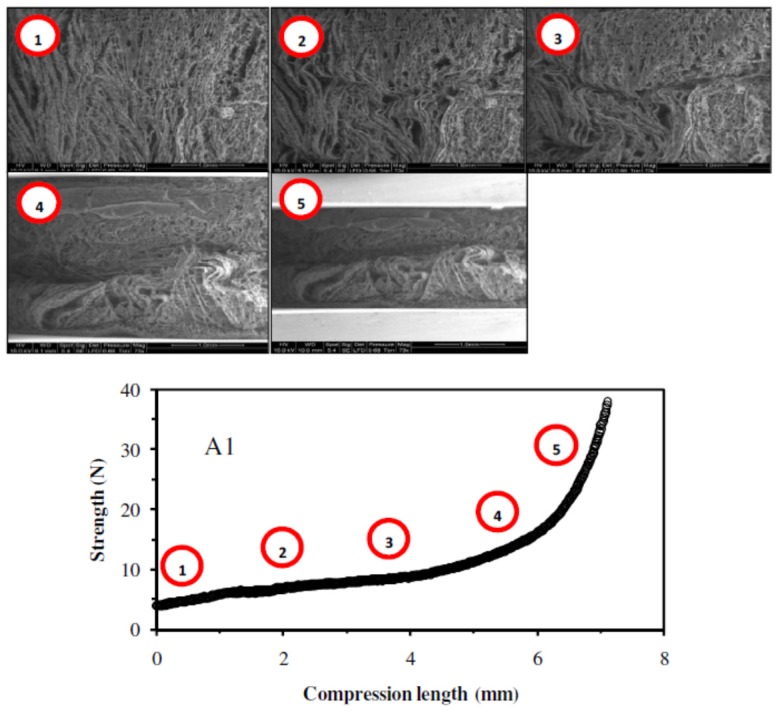
Compression test of **A1** catalytic material: SEM observations and evaluation of mechanical characteristics.

**Figure 4:**
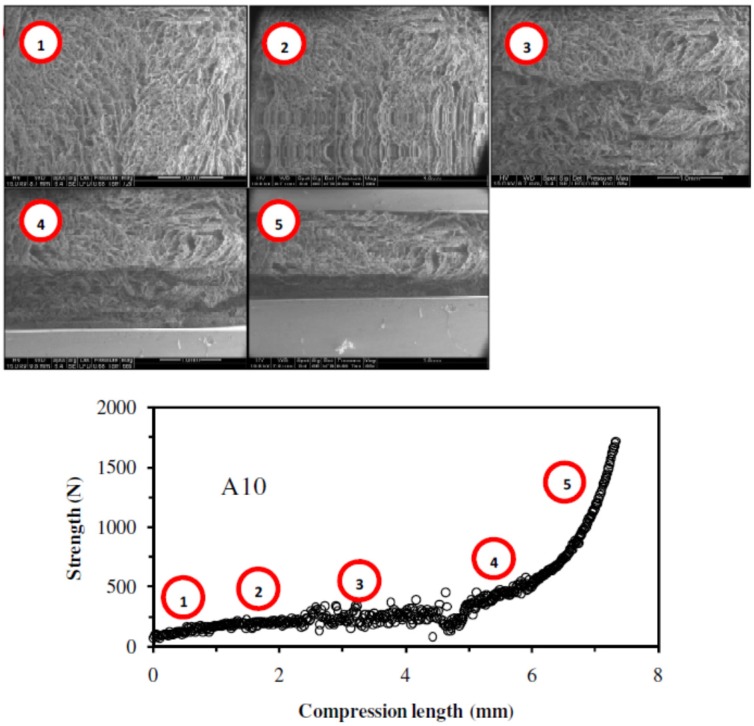
Compression test of **A10** catalytic material: SEM observations and evaluation of mechanical characteristics.

The elaboration of catalytic materials aimed at using them in continuous flow column reactor. With increasing the flow rate of the reaction, the catalytic materials were subjected to compression. To evaluate their mechanical resistance, compression studies of the catalytic materials were made using the SEM-Benco bench facilities. This procedure was not normalized but it allowed an approach to mechanical characteristics. Two catalytic materials were tested: a catalytic material prepared without mechanical strengthener (**A1** sample), and a catalytic material containing cellulose fibers (**A10** sample). The videos corresponding to the compression tests are available from the authors. Selected SEM pictures corresponding to different phases of the compression test are presented together with the mechanical tests in Figures 3 and 4.

The compression tests of the two catalytic materials were compared by the graph representing the strength (in N) as a function of the compression length (in mm). For both samples (**A1** and **A10**), the compression tests gave comparable profiles with two zones: (a) a first initial linear section corresponding to the increase of the strength with compression length, followed by (b) an exponential trend of the strength with compression length (Figures 3 and 4). The catalytic material prepared without mechanical strengthener (**A1** sample) could not undergo strength higher than 40 N, while the catalytic material prepared with cellulose fibers (**A10** sample), could undergo strength of 1750 N before crushing: the admissible strength was almost 50 times higher in the case of the catalytic material containing cellulose fibers. The cellulose fibers thus considerably increased the mechanical resistance to compression of the catalytic materials. This was consistent with the limited shrinking of the catalytic materials during the drying step when the cellulose fibers were included in the materials. Hence, the inclusion of cellulose fibers, as mechanical strengthener, drastically increased the mechanical properties of the catalytic materials.

### 2.2. Catalytic Properties of the Materials

As described in the previous section, among the different batches of catalytic materials, some showed poor stability in water limiting the possibility to use them for catalytic reaction (**A6–8** samples, Table 1). Thus, the catalytic properties of the materials were only studied for the materials that were stable in water (**A1–5** and **A9–10** samples).

The catalytic properties of the materials had been evaluated on the hydrogenation of 4-nitroaniline (4-NA) into *p*-phenylenediamine in the presence of formic acid as the hydrogen donor. Four parameters were measured to evaluate the activity of the catalytic materials: (a) the conversion yield, (b) the initial rate of the reaction, (c) the half-reaction time (HRT), and (d) the turnover frequency (TOF). The conversion yield was measured at complete conversion or after a maximum contact time of 60 min. The initial rate of the reaction was measured on the initial linear part of the curves (calculated for each reaction). The half-reaction time (HRT) corresponded to the time of reaction required for achieving the half of the conversion at 60 min. The turnover frequency (TOF) was measured at the HRT as the number of moles of substrate converted per mole of Pd and per minute. Table 3 reported the values of these catalytic parameters for the different materials. The influence of various parameters of elaboration of the catalytic materials (the freezing temperature, the M/G molar ratio, the gelation mode, the concentration of porogen agent, and the presence of cellulose fibers) were evaluated by the comparison of the conversion yields, the initial rates, the HRT and the TOF of the different catalytic materials.

**Table 3:** Catalytic properties of selected materials.

Material	Conversion yield (%) ^(a)^	time (min)	Initial rate (min^−1^)	Half-reaction time (min)	(mol 4-NA mol^−1^ Pd min^−1^)
A1	100	5	0.54	1.3	0.12
A2	100	6	0.51	1.5	0.14
A3	100	7	0.10	9	0.13
A4	90	3	0.11	14	0.28
A5	90	4	0.07	16	0.43
A9	70	7	0.04	26	0.29
A10	100	7	0.46	1.5	0.32
^(a)^ After a maximum reaction time of 60 min.

#### 2.2.1. Reproducibility of Experiments

**Figure 5:**
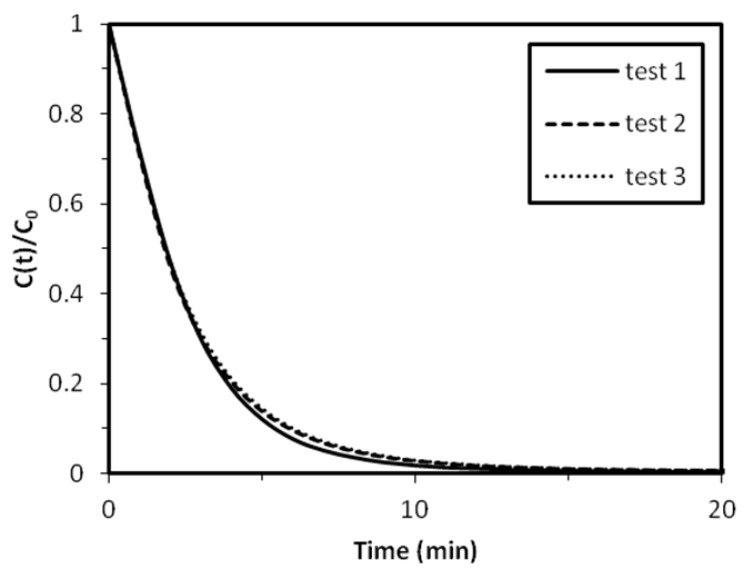
Reproducibility of the catalytic hydrogenation of 4-NA (Catalytic materials: **A1**).

In order to ensure the reproducibility of the experiments, the catalytic hydrogenation of 4-NA was evaluated three times under the same conditions with three catalytic materials from the same batch (**A1** sample) (Figure 5). The three experiments gave similar results, with only a small difference between 4 and 8 min. In this experiment, the maximum standard deviation was lower than 2.5%. The catalytic hydrogenation of 4-NA under these conditions was thus considered as reproducible. The reproducibility of the experiments was also punctually controlled with other catalytic materials.

#### 2.2.2. Influence of the Freezing Temperature

**Figure 6:**
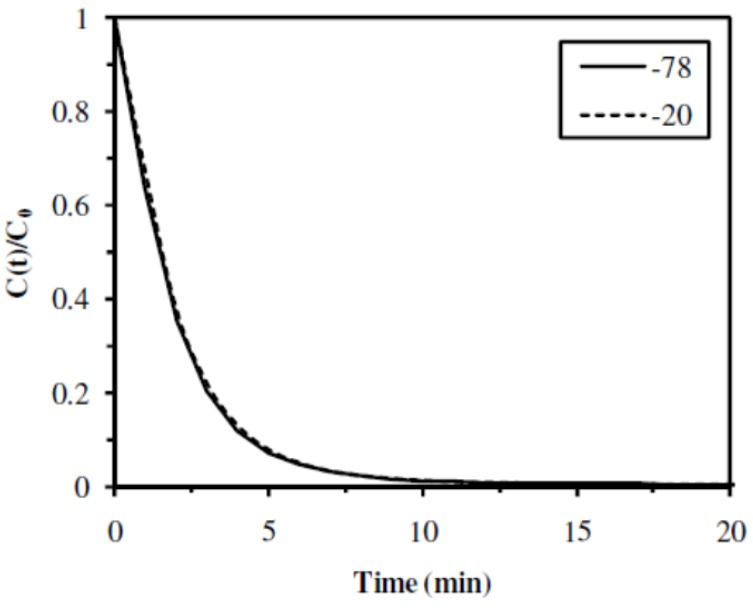
Influence of freezing temperature on the catalytic hydrogenation of 4-NA (Catalytic materials: **A1** (T: −78 °C), and **A2** (T: −20 °C)).

Two freezing temperatures were used for the elaboration of the catalytic materials: −78 °C (**A1** sample) and −20 °C (**A2** sample). As shown in Figure 6 and Table 3, the 4-NA hydrogenation reaction was not influenced by this parameter: both catalytic materials led to complete conversion yields, with similar initial rates (0.54 and 0.51 min^−1^), half-reaction times (1.3 and 1.5 min) and TOF (0.12 and 0.14 mol 4-NA mol^−1^ Pd min^−1^ for **A1** and **A2** samples, respectively). Even if the freezing parameter had an impact on the porous structure of the catalytic materials (as established in previous section and literature [26,27,28]), it had no influence on the catalytic properties. The porous structure of the catalytic material was opened enough, even at the freezing temperature of −20 °C, to make the opening of the network, a parameter of limited influence. That is the reason why, for large-scale production, a freezing temperature of −20 °C would be sufficient for the manufacturing of the catalytic materials.

#### 2.2.3. Influence of the M/G Molar Ratio

**Figure 7:**
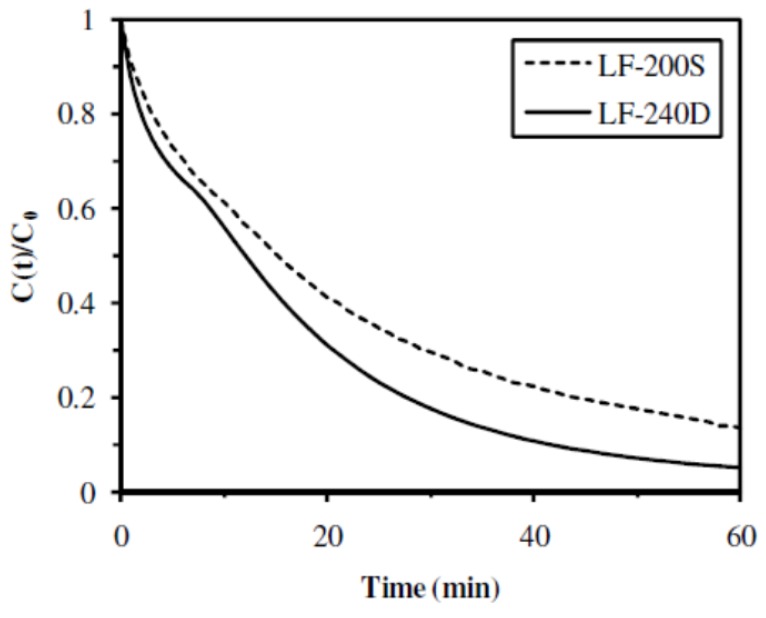
Influence of the M/G ratio of alginate on the catalytic hydrogenation of 4-NA (Catalytic materials: **A4** (Protanal LF-240D) and **A5** (Protanal LF-200S)).

Two catalytic materials prepared with alginates having different M/G molar ratio were studied for the hydrogenation of 4-NA: **A4** sample, rich in mannuronic acid groups (M/G molar ratio of 2.03), and **A5** sample, rich in guluronic acid groups (M/G molar ratio of 0.59). The change in the guluronic acid fraction slightly affected the hydrogenation kinetics of 4-NA: both catalytic materials reached 90% conversion yields at 60 min, but **A4** sample led to a slightly higher initial rate (0.11 min^−1^ compared to 0.07 min^−1^ for **A5** sample) and a slightly lower half-reaction time (14 min compared to 16 min for **A5** sample) (Figure 7 and Table 3). Nevertheless, as indicated in Table 2, the quantity of palladium immobilized in the two catalytic materials was slightly different: 20.7 and 15.3 mg Pd g^−1^ for **A4** and **A5** samples, respectively. The catalytic material prepared with alginate rich in mannuronic acid groups (**A4** sample) presented a TOF of 0.28 mol 4-NA mol^−1^ Pd min^−1^, lower than the catalytic material prepared with alginate rich in guluronic acid groups (**A5** sample) which had a TOF of 0.43 mol 4-NA mol^−1^ Pd min^−1^. Thus, there was a better rational use of the catalytic metal when the material was prepared with an alginate containing high guluronic acid fraction (**A5** sample, Protanal LF-200S).

#### 2.2.4. Influence of the Gelation Mode

**Figure 8:**
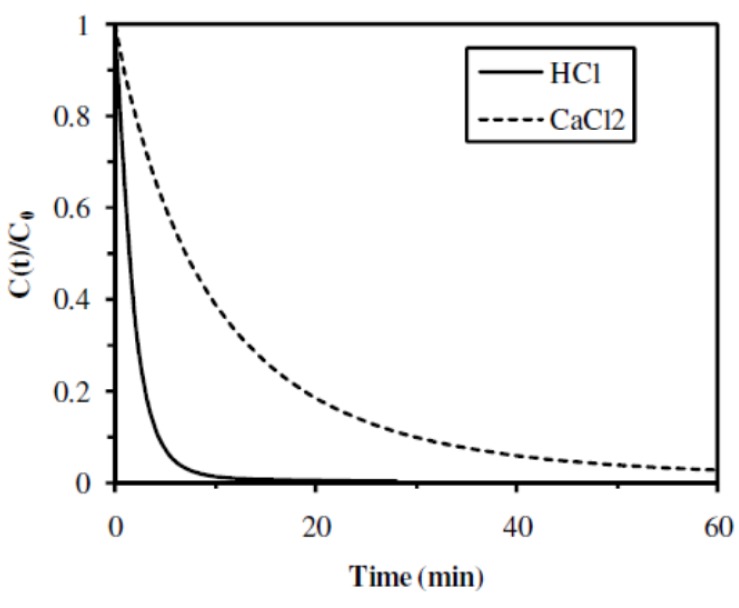
Influence of the type of gelation of the materials on the catalytic hydrogenation of 4-NA (Catalytic materials: **A1** (0.5 M HCl) and **A3** (0.4 M CaCl_2_)).

The influence of the gelation mode was studied comparing two catalytic materials: **A1** sample, prepared with gelation in HCl (alginic acid) and **A3** sample, prepared with coagulation in CaCl_2_ (Ca-alginate). Both samples led to complete conversion of the substrate with similar TOF: 0.12 and 0.13 mol 4-NA mol^−1^ Pd min^−1^ for **A1** and **A3** samples, respectively (Figure 8 and Table 3). Nevertheless, reaction kinetic was much higher in the case of the catalytic material prepared with alginic acid (initial rate of 0.54 min^−1^ and half reaction time of 1.3 min for **A1** sample) than in the case of the catalytic material prepared with Ca-alginate (initial rate of 0.10 min^−1^ and half reaction time of 9 min for **A3** sample). Catalytic reaction kinetic was higher with the catalytic material prepared with alginic acid (**A1** sample) than the catalytic material prepared with Ca-alginate (**A3** sample), but both samples led to good catalytic activity.

As described in previous sections, similar restrictions to mass transfer were observed for metal uptake when using the material prepared with Ca-alginate (Figure S2), and the gelation process also affected the internal structure of the catalytic materials (Figures S3 and AM5). The higher specific surface areas for alginate aerogels were obtained with the acidic gelation rather than with the Ca-ionotropic gelation [29]. Thus, the use of HCl gelation instead of calcium coagulation improved the porosity of the material and reduced the limitations in mass transfer for metal uptake and for 4-NA hydrogenation.

#### 2.2.5. Influence of the Alginate/NaHCO_3_ Ratio

**Figure 9:**
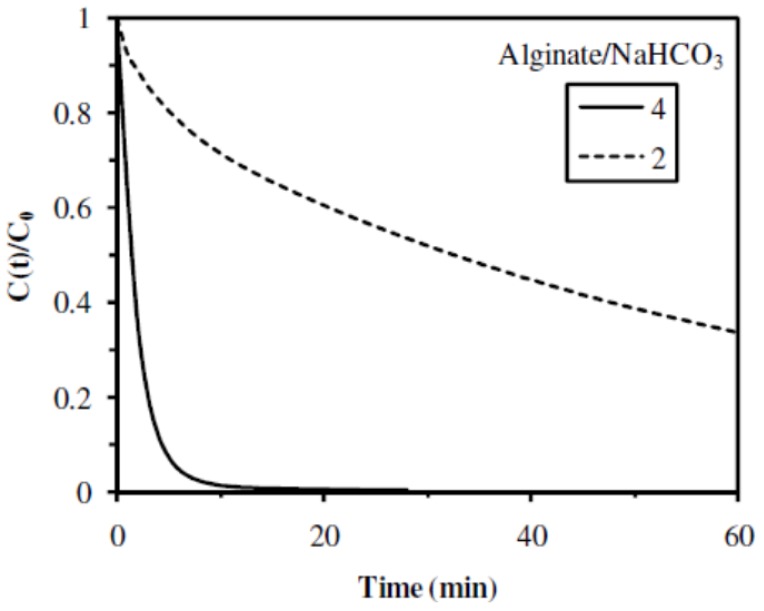
Influence of the Alginate/NaHCO_3_ mass ratio on the catalytic hydrogenation of 4-NA (Catalytic materials: **A1** (Ratio 4) and **A9** (Ratio 2)).

NaHCO_3_ salt acted as a porogen agent during the acidic gelation of the materials by causing the production of CO_2_ bubbles that might increase the porosity of the biopolymer matrix. As described in previous section, the use of a low amount of NaHCO_3_ (Alginate/NaHCO_3_ mass ratio of 8 in **A8** sample), did not allow to obtain a catalytic material stable in water. The influence of the concentration of porogen agent, in higher amounts, was studied by comparing the catalytic properties of two catalytic materials, prepared with the Alginate/NaHCO_3_ mass ratio of 4 in **A1** sample, and 2 in **A9** sample. A complete conversion of the substrate was obtained in 10–15 min with the catalytic material prepared with the Alginate/NaHCO_3_ mass ratio of 4 (**A1** sample), while, for the catalytic material prepared with the Alginate/NaHCO_3_ mass ratio of 2 (**A9** sample), even after 60 min of reaction, the conversion yield did not reach 70% (Figure 9 and Table 3). Indeed, the reaction kinetic was much higher in the case of the catalytic material prepared with the Alginate/NaHCO_3_ mass ratio of 4 (initial rate of 0.54 min^−1^ and half reaction time of 1.3 min for **A1** sample) than in the case of the catalytic material prepared with the Alginate/NaHCO_3_ mass ratio of 2 (initial rate of 0.04 min^−1^ and half reaction time of 26 min for **A9** sample). However, as indicated in Table 2, the amount of Pd immobilized was substantially lower in the catalytic material prepared with a high NaHCO_3_ level (22 mg Pd g^−1^ in **A9** sample), compared with the catalytic material prepared with a lower NaHCO_3_ level (62.8 mg Pd g^−1^ in **A1** sample). This difference in the amount of Pd immobilized in the materials should mainly explained the catalytic results presented in Table 3: even if the catalytic material containing a high NaHCO_3_ level (**A9** sample) led to a lower conversion yield, a higher TOF (0.29 mol 4-NA mol^−1^ Pd min^−1^) was obtained compared to the catalytic material containing a low NaHCO_3_ level (**A1** sample, with 0.12 mol 4-NA mol^−1^ Pd min^−1^). Thus, increasing the amount of NaHCO_3_ as porogen agent substantially increased the efficiency of the catalyst, but led to lower reaction kinetics.

#### 2.2.6. Influence of the Presence of Cellulose Fibers

**Figure 10:**
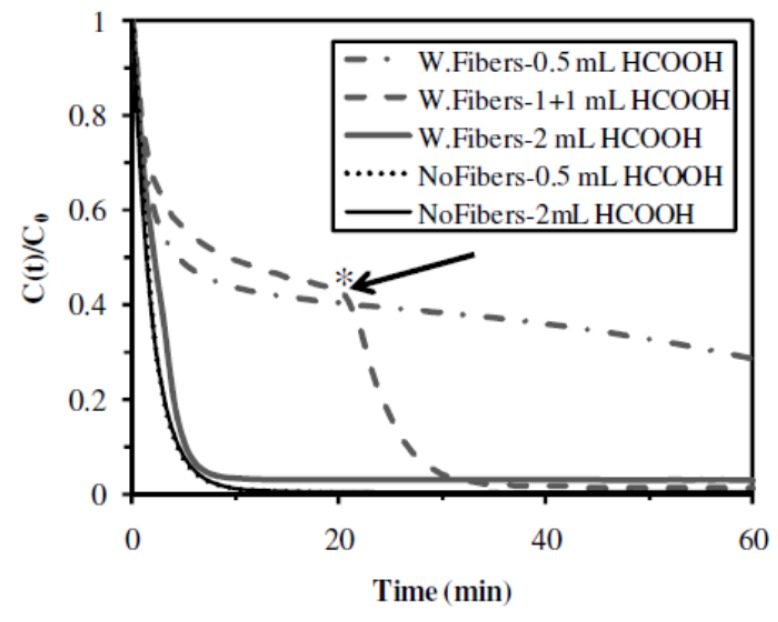
Impact of the presence of cellulose fibers and amount of formic acid on the catalytic hydrogenation of 4-NA (Catalytic materials: **A1** (NoFibers) and **A10** (W.Fibers); * supplementary addition of a 1-mL volume of formic acid).

The introduction of cellulose fibers, as mechanical strengthener in the catalytic material (**A10** sample), substantially changed the kinetics and the efficiency of the hydrogenation of 4-NA when compared to standard material (**A1** sample) (Figure 10). In the case of the catalytic material prepared without cellulose fibers (**A1** sample), a complete conversion of the substrate was obtained in 10 min, using a volume of hydrogen donor (formic acid) of 0.5 mL in 25 mL of substrate. Under similar concentration of hydrogen donor, when using the catalytic material containing cellulose fibers (**A10** sample), the conversion yield did not exceed 70% after 60 min of reaction. The initial rate (initial slope of the curve) was comparable to those obtained with standard material (**A1** sample), but after 15–20 min the reaction rates strongly reduced.

In the case of the catalytic material containing cellulose fibers (**A10** sample), the amount of hydrogen donor was then increased from 0.5 mL to 1 mL and a similar kinetic profile was obtained (Figure 10). However, when the concentration decay tended to stabilize, a new addition of formic acid (1 mL) was operated leading to the complete conversion of the substrate. While the concentration of formic acid did not influence the hydrogenation rate when using the standard material (**A1** sample), the introduction of cellulose fibers in the catalytic material required the use of higher amounts of hydrogen donor. When the hydrogen donor was added in a single drop (*i.e.*, 2 mL), at the beginning of the catalytic reaction, the kinetics of hydrogenation of both samples were similar: both catalytic materials led to complete conversion yields, but **A1** sample led to a slightly higher initial rate (0.54 min^−1^ compared to 0.46 min^−1^ for **A10** sample) and a slightly lower half-reaction time (1.3 min compared to 1.5 min for **A10** sample) (Figure 10 and Table 3). Nevertheless, as indicated in Table 2, the amount of Pd was substantially lower in the catalytic material containing cellulose fibers (18.9 mg Pd g^−1^ in **A10** sample) compared to the standard material (62.8 mg Pd g^−1^ in **A1** sample). Hence, the TOF was substantially better for the catalytic material containing cellulose fibers (0.32 mol 4-NA mol^−1^Pd min^−1^ for **A10** sample) than for the standard material (0.12 mol 4-NA mol^−1^ Pd min^−1^ for **A1** sample).

As a conclusion, in the case of catalytic materials containing cellulose fibers, higher amounts of hydrogen donor are required (4 times more). The possible absorption or adsorption of formic acid on the cellulose fibers could decrease the availability of the hydrogen donor for the catalytic reaction. However, it was impossible to find experimental evidence to support this hypothesis. Finally, the introduction of cellulose fibers substantially improved the mechanical resistance of the materials and its catalytic efficiency, but at the expense of increasing the amount of hydrogen donor to be delivered.

Worthy of note is that no leaching of the Pd on the outlet solutions was observed by ICP-AES analyses, which confirms the stability of all the catalytic materials tested.

### 2.3. Elaboration of Catalytic Materials for Specific Applications

All the analyses carried out in this work for the characterization of the catalytic materials allowed selection of different parameters depending on the desired properties of the materials.

In this study, the optimal catalytic material would combine a high porosity, a good mechanical stability, and a good catalytic activity. This optimal catalytic material could be elaborated with alginate with high guluronic acid groups at a concentration of 2% of biopolymer in solution, the addition of the porogen agent NaHCO_3_, the addition of cellulose fibers as mechanical strengthener, the elaboration of the materials by freezing at −20 °C, and the gelation with HCl.

For other specific applications, the elaboration of the catalytic material might be adjusted in order to obtain materials with suitable properties such as a high porosity, a good mechanical resistance to compression, a good stability in water, a high IL immobilization, high reaction kinetics or a high catalytic activity. Table 4 summarizes the different parameters to consider depending on the desired properties for the materials. Thus, alginate-supported ionic liquid and Pd materials could be elaborated with different and various properties.

**Table 4:** Parameters to consider depending on the desired properties for the materials.

Desired properties	Parameters for the elaboration of the materials
High porosity	No cellulose fibers
Good mechanical resistance to compression	Inclusion of cellulose fibers
Good stability in water	Alginate/NaHCO_3_ mass ratio of 2 or 4
High IL immobilization	No cellulose fibers
High reaction kinetics	HCl gelation
High catalytic activity	Inclusion of cellulose fibers (but increased amount of hydrogen donor)

## 3. Experimental Section

### 3.1. Materials

Three different alginate samples provided by Acros (A) and Protanal (LF 240D and LF 200S) were used for the synthesis of the biopolymer supports. Their viscosities in water (1% w/w concentration) were 485, 115 and 306 mPa·s, respectively. The respective M/G (mannuronic/guluronic acid) ratio of Protanal samples were determined by ^1^H NMR spectrometry. Protanal LF 240D was rich in mannuronic acid groups (*i.e.*, M/G: 2.03) while Pronatanal LF 200S was rich in guluronic acid groups (*i.e.*, M/G: 0.59). Two different ionic liquids (supplied by Cytec, Canada) were tested for the preparation of catalytic materials: Cyphos IL‑101 (*i.e.*, tetradecyl(trihexyl)phosphonium chloride) and Cyphos IL-111 (tetradecyl(trihexyl)phosphonium tetrafluoroborate). While Cyphos IL-101 is liquid at room temperature, Cyphos IL-111 has a melting temperature close to 37 °C. Those ILs were used as supplied (without any purification).

Other reagents are of analytical grade and were supplied by VWR, France (gelatin, HCl), Carlo Erba, Italy (NaHCO_3_, NaOH, H_2_SO_4_), Fluka AG, Switzerland (PdCl_2_, CaCl_2_) and J.T. Baker, France (Zn powder). Cellulose fibers were prepared from an Ahlstrom raw paper substrate (Pont-Evêque, France), a Roburflash-type paste prepared from resinous wood (long fibers) and with poor refinery level (flash), by dilacerations and rehydration.

### 3.2. Elaboration of the Catalytic Materials

#### 3.2.1. Elaboration of Highly Porous Monoliths (HPMs)

The preparation of highly porous monoliths (HPMs) was previously described for the elaboration of alginate-based catalytic materials [24]. A similar procedure was used for the elaboration of different alginate-supported ionic liquid and Pd HPMs. A solution of gelatin was prepared by dissolution in hot water (50 °C) under agitation to reach a 20% w/w concentration. Sodium hydroxide (0.25 g of a 10 M solution) and 2.5 g of a 20% w/w solution of gelatin were added to 1.25 g of ionic liquid. A solution of sodium alginate (2 or 3% w/w in water) was added to the former solution, to reach a final volume of 50 mL. The ionic liquid is hydrophobic and cannot be directly mixed with the aqueous alginate solution. The addition of the alkaline solution of gelatin contributes to compatibilize the IL with the aqueous alginate solution. The gelatin serves as a stabilizer of the IL-alginate mixture: it prevents the phase separation, at least for the duration of the synthesis. In the case of Cyphos IL-111, it was necessary to adapt the process of elaboration (maintaining the mixture at 50 °C using a bain-marie) to prevent the IL solidification. NaHCO_3_ was introduced in the mixture as a porogen agent, at different concentrations: with an Alginate/NaHCO_3_ mass ratio of 2, 4 or 8. In one sample, cellulose fibers, as natural mechanical strengthener, were added to the mixture, by dissolving first 0.5 g of cellulose from an Ahlstrom raw paper substrate in water solution (0.5% w/w in water), before adding sodium alginate and finally mixing with the others reactants. The final content of cellulose fibers in the material (after material drying) was about 7% w/w (dry state).

The next step consisted in the distribution of the viscous mixture containing alginate and IL in moulds (plate formed of cylindrical holes of 10 mm height and 10 mm diameter) that were maintained for 1 h at either −78 °C or −20 °C as freezing temperature. The frozen cylinders were then removed from the moulds and dropped in a gelation bath made of either a 0.5 M HCl solution or a 0.4 M CaCl_2_ solution at the temperature of 1 °C (container surrounded by crushed ice) for 10 h. Finally, the materials were rinsed with tap water and freeze-dried with an Alpha 1–4 LD freeze-dryer (Christ, Sigma).

The alginate-supported IL HPMs obtained under the previous conditions for the different batches, named **A1** to **A10**, are reported in Table 1.

#### 3.2.2. Metal Immobilization

The sorption of palladium in the previous alginate-supported IL materials was operated by leaving the materials for 2 days in a 150 ppm solution of palladium chloride at pH 1 (controlled with HCl solution), under soft agitation (120 rpm). The reduction of the palladium immobilized in the materials was operated by *in situ *produced hydrogen: the Pd(II)-materials were immersed in a 0.5 M H_2_SO_4_ solution in the presence of zinc powder for 3 days at 30 °C under agitation, to allow a complete palladium reduction. The Pd(0)-materials were rinsed with water and dried by freeze-drying.

### 3.3. Characterization of Catalytic Materials

The morphology (porosity) and the distribution of IL and Pd in the materials were determined with a Scanning Electron Microscope coupled with Energy Dispersive X-ray analysis (SEM-EDX). SEM observations were performed using an Environmental Scanning Electron Microscopy (ESEM) Quanta FEG 200, equipped with an OXFORD Inca 350 Energy Dispersive X-ray microanalysis (EDX) system. The SEM observations were performed on the cross-section of the catalytic materials (obtained by cutting with a thin-slice cutter). The use of environmental SEM allowed the direct observations of materials, without previous metallization of the samples. The topography of the samples was observed using secondary electron flux while the backscattered electrons were used for the identification and localization of heavy metals at the surface of the materials (by phase contrast). SEM-EDX facilities were used for the analysis of specific zones at the surface of the catalytic materials.

Mechanical tests (resistance to compression) were performed using the facilities of the SEM equipped with a mechanical testing unit (Benco symmetrical bench) on the catalytic materials (10 mm height and diameter). The catalytic material, fixed inside the SEM, was submitted to a compression test. The SEM facilities allow to follow the force measured on the sensors and the mechanical degradation of the catalytic material submitted to a 5.3 kN compression strength. A video reporting a series of microphotograph of the compression area could be recorded.

The amount of IL and Pd immobilized on the catalytic materials were determined by mineralization of the materials (in triplicate [24]) followed by palladium, phosphorus and boron analyses with an ICP-AES spectrophotometer (Inductively Coupled Plasma with Atomic Emission Spectrometer) Jobin-Yvon Activa-M (Jobin-Yvon, Longjumeau, France). The P element was used as a tracer of the ionic liquid cation (Cyphos IL-101 and Cyphos IL-111), and B element as a tracer of Cyphos IL-111 anion.

The free volume of the materials was determined using a pycnometer. The void volume of the catalytic materials was close to 90%.

### 3.4. Catalytic Reaction

The study of the catalytic activity of the materials was carried out on the hydrogenation of 4-nitroaniline (4-NA) to *p*-phenylenediamine in the presence of formic acid as the hydrogen donor. This simple test-reaction was used to compare the catalytic efficiency of the different supports using the optimized reaction conditions obtained in a previous study [24]. 25 mL of a solution of 4-NA (25 mg L^−1^) was prepared. 0.5 mL of formic acid (99%, w/w), was added to the substrate solution under agitation using a magnetic stirrer. The temperature of the reactor was maintained at 25 °C during the experiment by a temperature-controlled box. The catalytic material was immobilized in a column, blocked by inert synthetic foam (Figure S10, Supplementary Information). The column was fed by a peristaltic pump and the solution was continuously recirculated through column at the flow rate of 15 mL min^−1^. An auxiliary recirculation loop was used for the analysis of the solution: the solution was circulated at the flow rate of 12 mL.min^−1^ through a circulating-cuvette to measure on-line the absorbance of the substrate at 381 nm with a Shimadzu UV-1650PC spectrophotometer. The measurement of the absorbance at 381 nm allowed to evaluate the concentration of 4-NA.

The efficiency of the different catalytic materials was compared by the conversion yield (at complete conversion or after a maximum contact time of 60 min), by the half-reaction-time (HRT, the time of reaction required for achieving the half of the conversion at 60 min) and by the TOF (turn-over-frequency, mol 4-NA mol Pd^−1^ min^−1^) measured at the HRT. The TOF was determined taking into account the mass balance on substrate concentration, the reaction time and the amount of Pd in the catalytic material.

## 4. Conclusions

This study showed that alginates could be used to immobilize ionic liquids (such as phosphonium cation ILs) in the form of highly porous monoliths (HPMs) to prepare catalytic materials. The binding of Pd(II) on the alginate-supported IL materials through an ion exchange mechanism, followed by metal reduction, led to efficient catalytic materials for 4-nitroaniline hydrogenation in the presence of formic acid as the hydrogen donor. Designing the biopolymer support in the form of HPMs allowed to test the properties of the catalytic materials for the hydrogenation of 4-nitroaniline in column systems. A series of experimental conditions (the M/G ratio of alginate, the biopolymer concentration, the freezing temperature, the type of ionic liquid, the concentration of porogen agent, the type of coagulating agent, and the presence of cellulose fibers as natural mechanical strengthener) were set up for the elaboration of the catalytic materials in order to study their impact on structural properties (pore size, pore form), the distribution of metal particles, the mechanical properties of the HPMs and the catalytic properties of the materials.

The use of alginate as immobilizing matrix for IL and Pd showed good results in terms of mechanical properties (especially when cellulose fibers, as mechanical strengtheners, were incorporated in the materials), metal sorption properties and catalytic efficiency.

The freezing temperature affected structural properties of the materials but not catalytic performance. The properties of alginate (M/G molar ratio) influenced catalytic performance. The presence of cellulose fibers improved both mechanical properties and the catalytic efficiency, but at the expense of increasing the concentration of the hydrogen donor required for the hydrogenation reaction.

An optimal catalytic material could be elaborated with alginate with high guluronic acid groups at a concentration of 2% of biopolymer in solution, the addition of the porogen agent NaHCO_3_, the addition of cellulose fibers as mechanical strengthener, the elaboration of the materials by freezing at −20 °C, and the gelation with HCl. This optimal HPM combines a high porosity, a good mechanical stability, and a good catalytic activity.

This study also provides support for the elaboration of alginate-supported ionic liquid and Pd materials, highlighting the parameters to be considered, depending on the desired properties of the materials.

## Supplementary Materials

Supplementary File 1
