# Supplementary material for: Study of Alginate-Supported Ionic Liquid and Pd Catalysts
Source: Nanomaterials (Basel). 2012 Jan 11;2(1):31–53. doi: 10.3390/nano2010031 (PMC5327881; doi:10.3390/nano2010031)

Nanomaterials **2012**, *2*, 31-53; doi:10.3390/nano2010053

OPEN ACCESS

nanomaterials

ISSN 2079-4991

www.mdpi.com/journal/nanomaterials

Article

Study of Alginate-Supported Ionic Liquid and Pd Catalysts

Claire Jouannin [1,2], Chloë Vincent [1], Isabelle Dez [2], Annie-Claude Gaumont [2], Thierry Vincent [1] and Eric Guibal [1,*]

[1] Ecole des Mines d'Alès, Laboratoire Génie de l'Environnement Industriel, Equipe Interfaces Fonctionnalisées pour l'Environnement et la Sécurité, 6, Avenue de Clavières, F-30319 ALES Cedex, France; E-Mails: claire.jouannin@ensicaen.fr (C.J.); chloe_vincent@live.fr (C.V); thierry.vincent@mines-ales.fr (T.V.)

[2] Laboratoire de Chimie Moléculaire et Thioorganique, UMR CNRS 6507, INC3M, FR 3038, ENSICAEN & Université de Caen, 14050 Caen, France; E-Mails: isabelle.dez@ensicaen.fr (I.D.); annie-claude.gaumont@ensicaen.fr (A.-C.G.)

* Author to whom correspondence should be addressed; E-Mail: eric.guibal@mines-ales.fr; Tel.: +33-(0)-466782734; Fax: +33-(0)-466782701.

Supplementary Information

Figure S1. Comparison of Pd(II) uptake kinetics for **A1** and **A3** samples (pH 1).

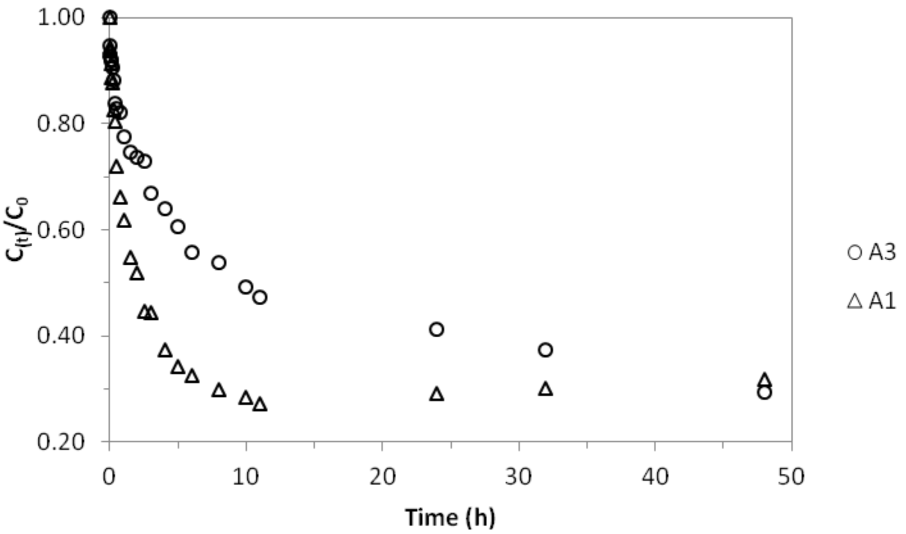

Figure S2. Comparison of Pd(II) sorption isotherm for **A1**, **A2** and **A3** samples (pH 1).

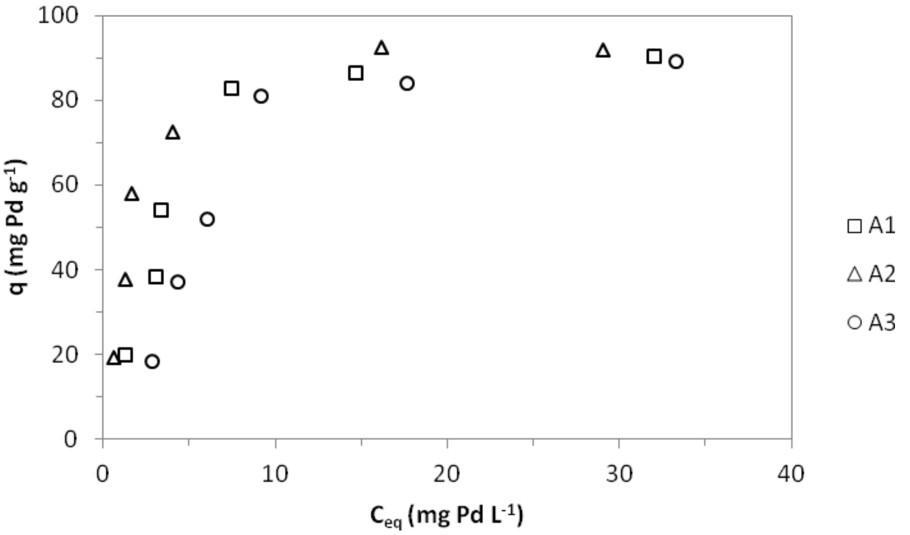

Figure S3. SEM photographs of **A1** catalytic material (bar scale: (**a**) and (**b**) 1 mm, (**c**) 100 µm and (**d**): 20 µm; (**a**): secondary electrons for morphology observations; (**b-d**) backscattered electrons for phase contrast determination of heavy elements).

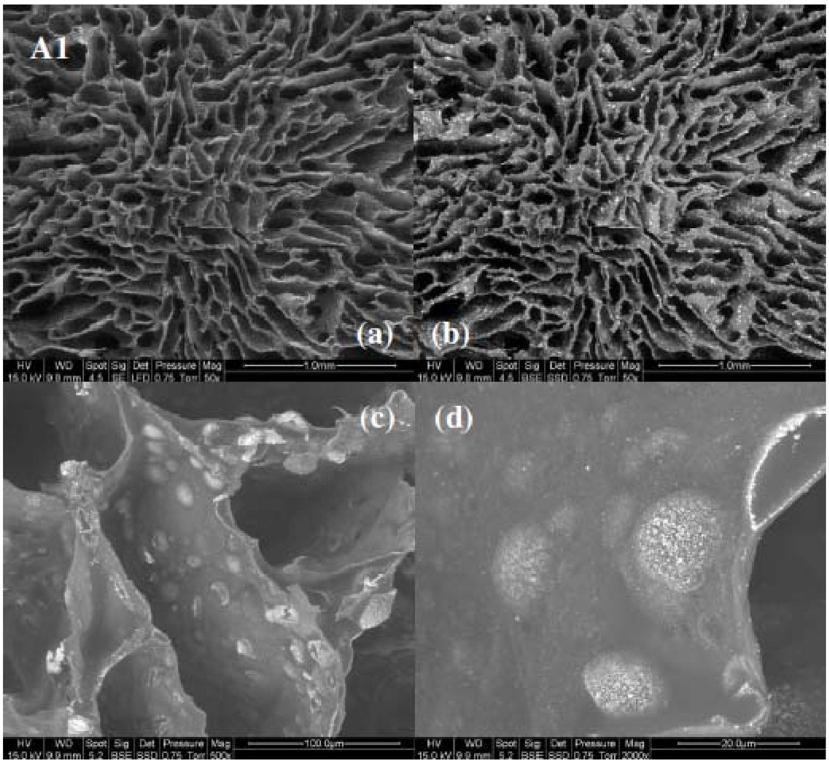

Figure S4. SEM photographs of **A2** catalytic material (bar scale: (**a**) and (**b**) 1 mm, (**c**) 100 μm and (**d**): 20 μm; (**a**): secondary electrons for morphology observations; (**b-d**) backscattered electrons for phase contrast determination of heavy elements).

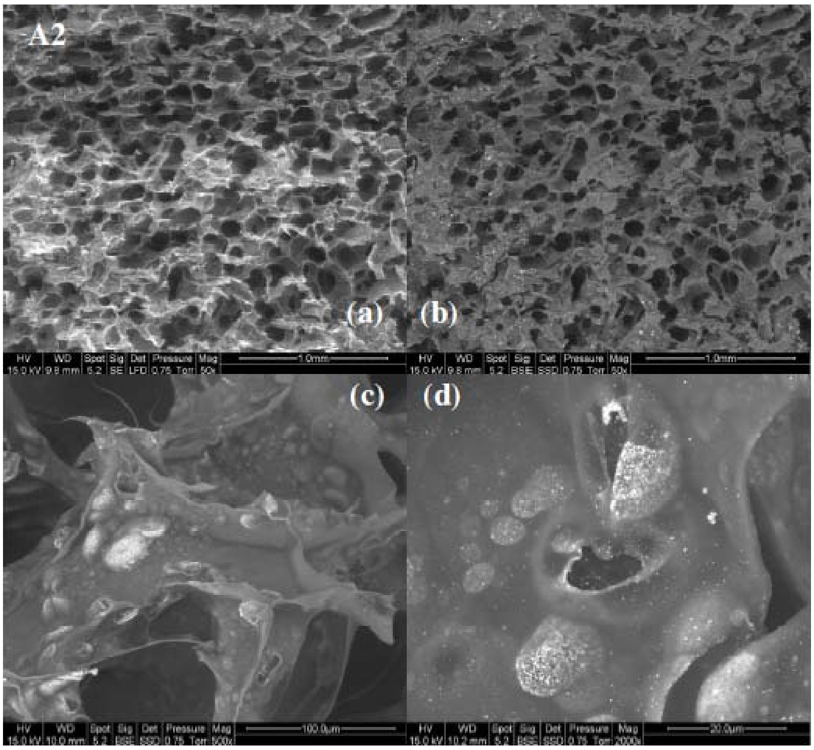

Figure S5. SEM photographs of **A3** catalytic material (bar scale: (**a**) and (**b**) 1 mm, (**c**) 100 μm and (**d**): 20 μm; (**a**): secondary electrons for morphology observations; (**b-d**) backscattered electrons for phase contrast determination of heavy elements).

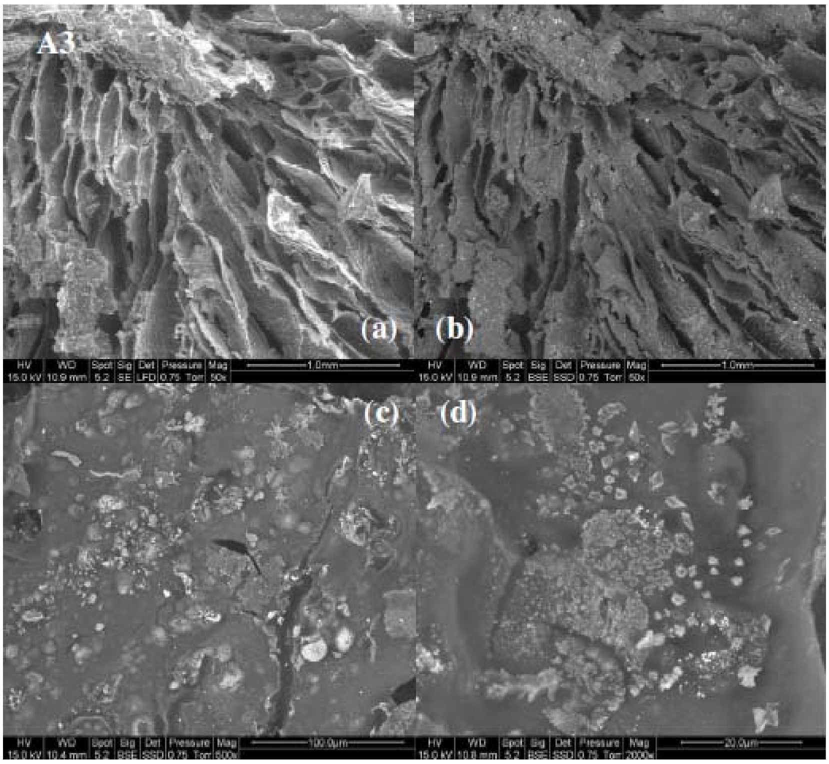

Figure S6. SEM photographs of **A4** catalytic material (bar scale: (**a**) and (**b**) 1 mm, (**c**) 100 µm and (**d**): 20 µm; (**a**): secondary electrons for morphology observations; (**b-d**) backscattered electrons for phase contrast determination of heavy elements).

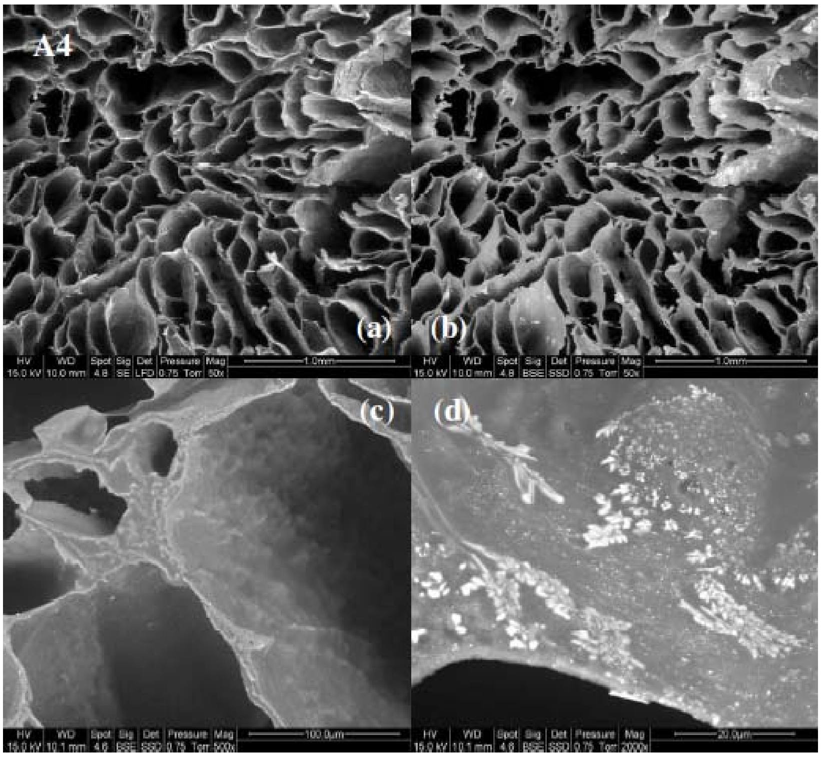

Figure S7. SEM photographs of **A5** catalytic material (bar scale: (**a**) and (**b**) 1 mm, (**c**) 100 µm and (**d**): 20 µm; (**a**): secondary electrons for morphology observations; (**b-d**) backscattered electrons for phase contrast determination of heavy elements).

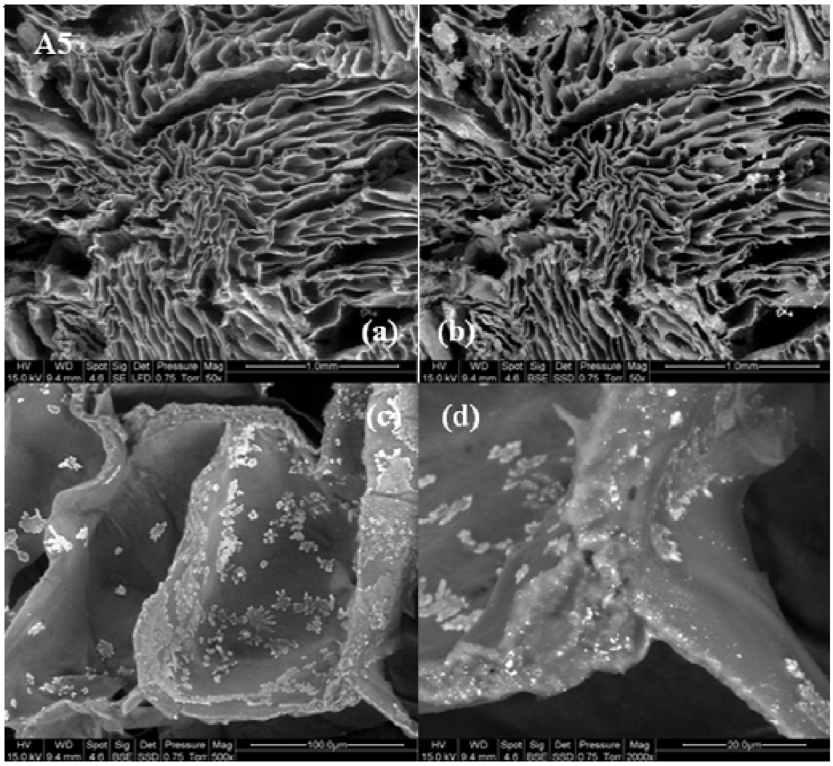

Figure S8. SEM photographs of **A9** catalytic material (bar scale: (**a**) and (**b**) 1 mm, (**c**) 100 μm and (**d**): 20 μm; (**a**): secondary electrons for morphology observations; (**b-d**) backscattered electrons for phase contrast determination of heavy elements).

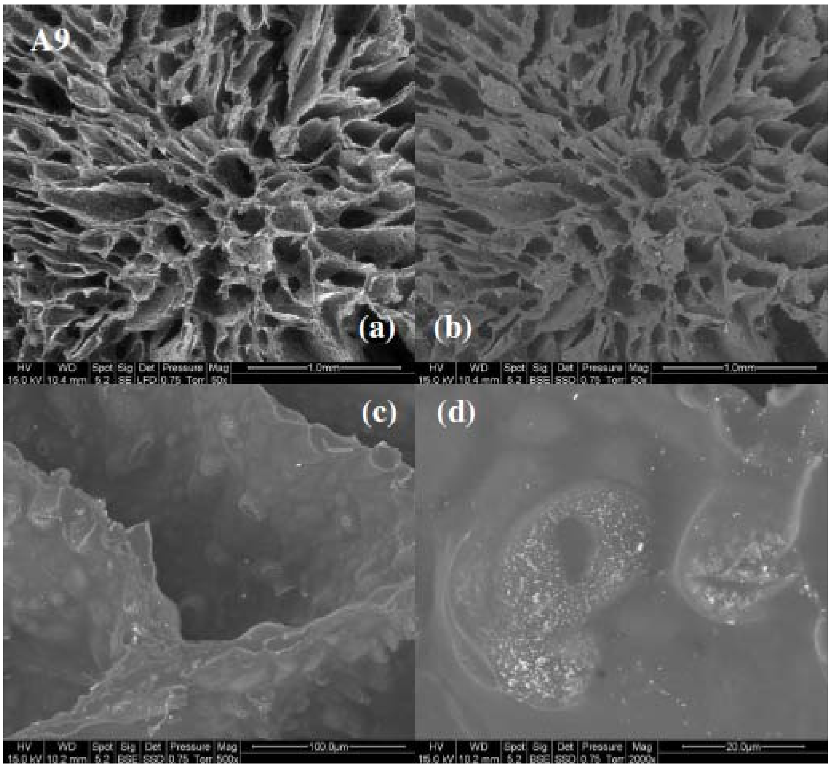

Figure S9. SEM photographs of **A10** catalytic material (bar scale: (**a**) and (**b**) 1 mm, (**c**) 200 μm and (**d**): 20 μm; (**a**): secondary electrons for morphology observations; (**b-d**) backscattered electrons for phase contrast determination of heavy elements).

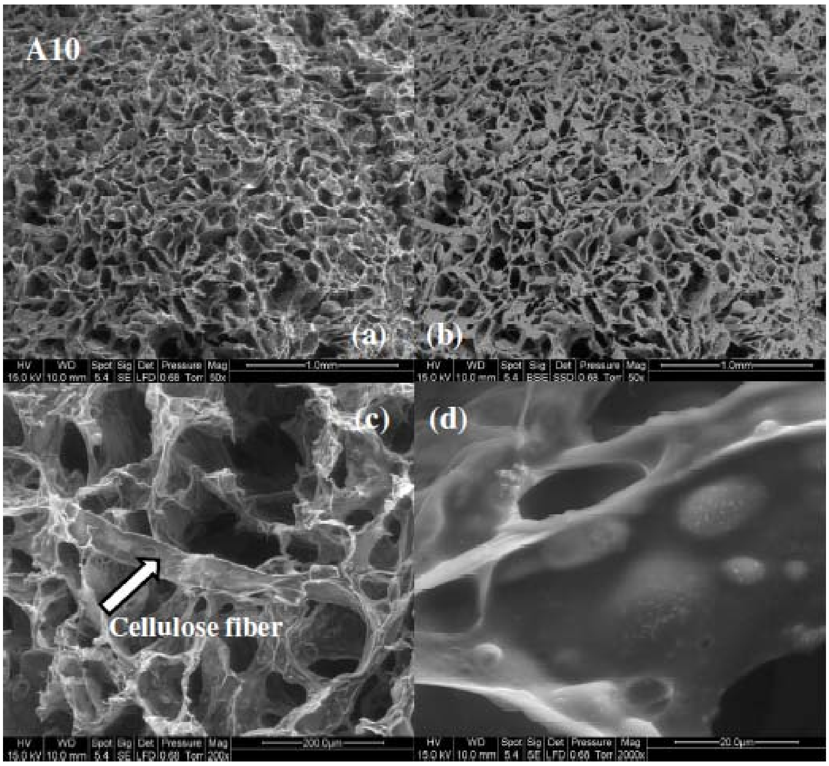

Figure S10. Experimental set-up for the investigation of catalytic hydrogenation of 4-NA using alginate-supported IL and Pd materials.

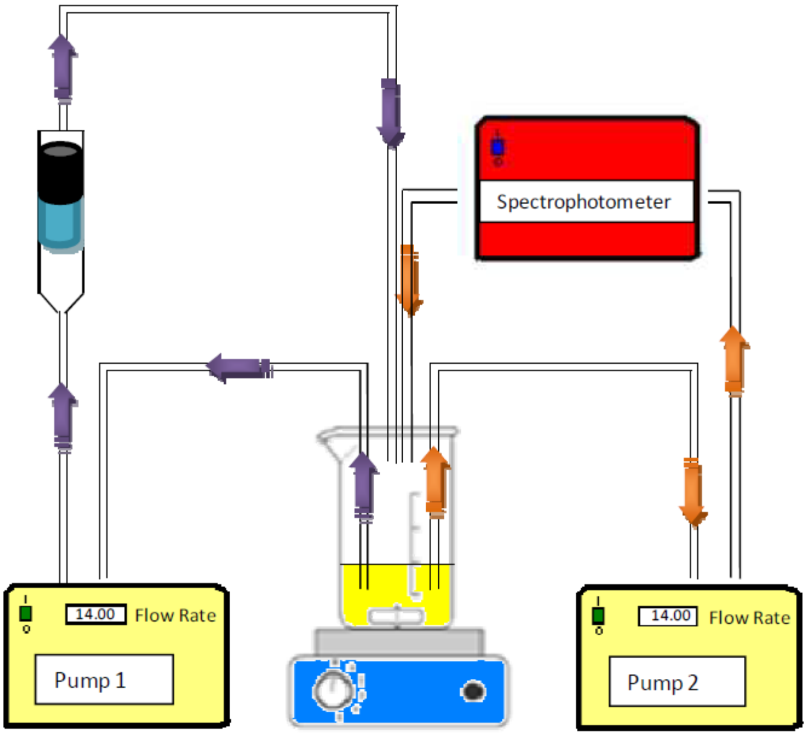

Supplement: Supplementary File 1 [file nanomaterials-02-00031-s001.pdf]
